# Carbon dioxide removal through ecosystem restoration: Public perceptions and political participation

**DOI:** 10.1007/s13280-024-02063-y

**Published:** 2024-08-23

**Authors:** Annegret Kuhn, Christine Merk, Andrea Wunsch

**Affiliations:** 1 Center for Ocean and Society, Kiel University, Christian-Albrechts-Universität zu Kiel, Neufeldtstr. 10, 24118 Kiel, Germany; 2 Kiel Institute for the World Economy, Kiellinie 66, 24105 Kiel, Germany

**Keywords:** Climate change mitigation, CO_2_ removal, Ecosystem restoration, Political trust, Political participation, Public perception

## Abstract

We compare public perceptions of restoring different ecosystems to increase CO_2_ uptake in Germany, through focus groups and a general population survey. Among focus group participants forests were highly popular, peatlands evoked negative associations, and seagrass was largely unknown. Nevertheless, the restoration of all ecosystems was viewed positively. We contrast these reactions to those of survey respondents who had not received additional information on restoration. They voiced narrower, less diverse opinions centering around afforestation. Further, focus group participants preferred expert-led restoration decisions, citing low trust in politicians’ technical competence. Contrary to common policy recommendations, also beyond the German context, participants did not emphasize the need of citizen participation and were not strongly concerned about land use conflicts or compensation of affected user groups. The results imply that the public underestimates the political complexity of negotiation processes in ecosystem governance, which are becoming increasingly relevant in the international policy landscape.

## Introduction

Ecosystem restoration increasingly features in many countries’ Nationally Determined Contributions (NDCs) as a measure to support climate change adaptation and mitigation. Mitigation measures include the reduction of emissions by protecting ecosystems that store carbon such as forests, peatlands, or seagrass meadows and by restoring them. The majority of NDCs mention forests, while coastal ecosystems and wetlands are included less frequently (Seddon et al. 2020; UNEP and IUCN 2021). Furthermore, many NDCs focus on managing and restoring rather than on protecting these ecosystems (UNEP and IUCN 2021). Protection helps to maintain and potentially increase the current carbon stock, whereas restoration would reduce land-based emissions and remove additional CO_2_ from the air, i.e., achieve carbon dioxide removal (CDR). However, large-scale ecosystem restoration faces several potential conflicts. Land use competition can arise, as restoration projects might compete with agricultural needs and social conflicts can arise where local communities depend on the land for their livelihoods and their use of ecosystem services is affected. Economic constraints also play a significant role, as restoration projects require substantial investment (IPCC 2019). Addressing these conflicts requires taking into account stakeholders’ perceptions and interests to balance environmental, social, and economic interests, which will be highly relevant for the feasibility of policy initiatives for large-scale restoration such as the Action Program for Natural Climate Protection in Germany (Deutscher Bundestag 2023), the planned EU Nature Restoration Law, or the Kunming-Montreal Global Biodiversity Framework (United Nations Conference of the Parties 2022).

As one of the first studies, this paper contrasts perceptions of forests, peatlands, and seagrass meadows in the general population and studies how these perceptions influence their relative preferences for the restoration of these ecosystems as a climate protection measure. Based on six in-depth focus group discussions in Germany and contrasted by results from a general population survey, we analyze participants’ reasoning for and against human interventions with the aim to restore. In doing so, we add to the limited body of literature on landscape preferences adding insights for upcoming debates about large-scale restoration for carbon storage and biodiversity. Further, we particularly glean new findings on public perceptions of one so far sparsely investigated ecosystem, namely seagrass (Cullen-Unsworth et al. 2014; de la Torre-Castro et al. 2014; Nordlund et al. 2018). Moreover, we also contribute to the existing research by adding novel findings on public perceptions of political participation in ecosystem governance. In particular, we find that contrary to prevailing policy recommendations on public participation, broad citizen participation was not perceived as necessary or desired.

Comparing the perceptions of forests, peatlands, and seagrass, we expect that a stronger charisma will lead to a higher valuation of an ecosystem compared to less charismatic alternatives and to stronger support for its restoration (Orth et al. 2006; Gobster et al. 2007; Byg et al. 2023). But we go beyond charisma and argue that it is related to the level of awareness of and experience with the ecosystem in question. There are connections between conservation/biodiversity values, familiarity, scenic beauty, recreational services, and the perceived attractiveness or valuation of an ecosystem (Han 2007; Kiley et al. 2017). To the best of our knowledge, only Han (2007) and Kiley et al. (2017) have explicitly compared preferences for different terrestrial ecosystems based on their characteristics and services.

A generally positive perception of forests is widely acknowledged, with existing literature referring to it as charismatic mega-flora (Hall et al. 2011), frequently depicting forests as “the symbol for Nature” (Lehmann and Schriewer 2000) and documenting public appreciation often linked to recreational activities (Meyer et al. 2019; Rathmann et al. 2020). Further, Racevskis and Lupi (2006) found a strong concern among residents of Michigan, who do not depend on timber, about maintaining recreational opportunities when forest management is changed. More than three-quarters of Germans visit a nearby forest at least once a year to enjoy the recreational services provided by this ecosystem (Elsasser and Weller 2013). In China’s Ansai region, Tan et al. (2021) found that forests were perceived as most important, whereas water bodies were considered least important for providing cultural ecosystem services.

For Scottish peatlands, Byg et al. (2017) and Faccioli et al. (2020) reported certain negative connotations with wasteland, although the landscape was also valued as a part of Scottish identity. Similar to the case of forests, personal experiences contribute to shaping positive perceptions of peatlands as natural landscapes, as Flint and Jennings (2022) found by analyzing user-generated reviews from three peatland sites in England. Overall, the non-market benefits of peatland restoration in Scotland, including carbon storage, were found to enhance welfare (Glenk and Martin-Ortega 2018).

Nordlund et al. (2018) and Ruiz-Frau et al. (2018) found low levels of knowledge about seagrass and its services among the public. This may have been due to the particularly low media coverage identified by, e.g., Fernández et al. (2022) for Northern Spain, and may also have been related to a perception of seagrass as *“uninteresting”* and *“non-charismatic”* (Duarte et al. 2008; Jefferson et al. 2014). Personal experiences with seagrass are often negative when it washes ashore as beach wrack (Fernández et al. 2022), which is perceived as a nuisance (Ruiz-Frau et al. 2018) especially as it is lumped together with other kinds of decaying biomass at the beach that smells bad, even though decaying seagrass itself does not emit a strong odor (Weinberger et al. 2021).

Ecosystem restoration in the context of carbon dioxide removal, increases the scale in terms of the size of the area and the extent of the intervention into current ecosystems strongly. It also puts them in the context of a broader portfolio of approaches including also direct air capture or bioenergy with geological storage, or alkalinity enhancement. To date, research on public perceptions of CDR has often focused on the differences between approaches that are on opposite ends of a spectrum of perceived naturalness, and have consistently shown that perceived naturalness is a strong predictor of positive perceptions and acceptability of specific approaches (Wolske et al. 2019; Bellamy and Osaka 2020; Bellamy 2022). In many cases, afforestation is presented as a “nature-based solution” in contrast to more technological CDR options such as direct air capture (Jobin and Siegrist 2020; Wenger et al. 2021; Merk et al. 2023). Afforestation is seen as being, e.g., benign and natural, even if it involves a massive intervention or replacement of other ecosystems (Braun et al. 2018). Nawaz et al. (2023) and Veland and Merk (2021) identified a similar relationship between coastal ecosystem restoration and ostensibly more technological approaches like ocean alkalization. Perceptions of the restoration of different terrestrial and marine ecosystems in general and for CDR in particular are, however, rarely examined together and compared, even though the ecosystems differ in terms of, e.g., land use, co-benefits, costs, or potential conflicts of use (Brown 2020; Bodin et al. 2022). These factors are, however, likely to influence perceptions, together with general valuations of the ecosystems (Racevskis and Lupi 2006; Tolvanen et al. 2013; Börger and Piwowarczyk 2016). For example, Bellamy (2022)—in a rare exception that also compares different ecosystem-based options—found that peatland restoration was evaluated most favorably in the UK compared to other methods such as afforestation, wood in construction, or direct air capture.

Turning to the actual implementation of ecosystem restoration, existing studies have generally identified a quite low level of trust in the capacity of politicians to adequately handle environmental governance (Hynes et al. 2014). In the *Special Eurobarometer on Climate Change* (European Commission 2021), a large majority of respondents in Europe (including Germany) were skeptical about government actions to tackle climate change. In similar vein, Gkargkavouzi et al. (2020) and Hawkins et al. (2016) found quite negative public attitudes toward marine environmental governance in European countries. There seem to be substantial differences in the degrees of political trust between countries and between different levels of governance, e.g., local, national, or supranational (Hawkins et al. 2016; Gkargkavouzi et al. 2020; European Commission 2021); in addition, perceptions of ecosystem governance may depend on the general level of trust in political institutions (Braun et al. 2018; Bertram and Merk 2020).

Instead of trusting politicians, some studies have found that the public appear to trust in scientists and their expertise to adequately manage the marine environment (Hynes et al. 2014). Furthermore, connections to the government apparently taint the public’s trust in scientists, as researchers at universities are more trusted compared to those who work for the government (Gelcich et al. 2014). This suggests that people suspect scientists who work for the government of being entangled in political processes that are generally considered to be non-transparent and characterized by the dominance of self-interested politicians.

Involving the public, i.e., stakeholders or rights holders, is considered essential to successful ecosystem management projects, not only because it potentially affects the local population’s livelihoods and the local use of ecosystem services (Gobster and Hull 2000; Robinson 2011; Bennett 2016; Quevedo et al. 2020). Furthermore, implementation depends on local legitimacy and cooperation to enforce, e.g., access restrictions (Paletto et al. 2015; Byg et al. 2017; Merk et al. 2022). However, when asked to assess different CDR options, participants in a general population survey in the UK rarely chose political feasibility and social acceptability as one the three most relevant assessment criteria, prioritizing effectiveness, environmental impacts, and safety instead. Furthermore, they considered the political feasibility and social acceptability to be highest for peatland restoration and afforestation compared to, e.g., Direct Air Carbon Capture and Storage (DACCS) (Bellamy 2022).

In the following, we first describe the focus group setup, where we aim for an in-depth discussion of the restoration of forests, peatlands, and seagrass meadows. Second, we describe the general population survey, which contrasts the more context-dependent assessments from the focus groups with perceptions of participants with very little context information. The results section provides a systematic overview of the six focus group discussions and the answers from the “uninformed” survey respondents. In the final section, we synthesize the results, put them in a broader context, and discuss limitations and avenues for future research.

## Materials and methods

### Focus groups and general population survey

A 2-h online format using Zoom, a videoconferencing application, was used instead of in-person focus groups due to COVID-19-related restrictions. Six focus groups with 4–5 participants each and 29 in total were held in February 2022. The participants were recruited by the panel provider IPSOS and selected on the basis of their age, gender, and level of education, with a view to representing a diverse range of backgrounds. We excluded participants working in the fields of climate and environment to guarantee a discussion among people with similar levels of expertise. Further, we only included people who had claimed to have an affinity for nature and who had previously visited the German coast to ensure that they would be interested in actively engaging in the discussions.^1^ The groups were clustered by region: two focus groups consisted of participants living close to the German Baltic Sea coast (15–20 km from the coast, referred to as ‘coast’), two groups of participants hailed from larger cities (> 100 000 inhabitants, one in northern Germany, one in southern Germany, collectively referred to as ‘urban’), and two groups lived in smaller cities or villages (< 15 000 inhabitants, referred to as ‘rural’). Each participant received 50 euros. A professional moderator led the discussions and one of the authors provided information on ecosystem restoration using graphics, pictures, and overview tables. She also answered participants’ questions. The information was meant to create a common basis for the subsequent discussion, which followed an interview guide and consisted of four thematic sections.

Section “Introduction” began with questions on the participants’ relationship with nature in general, their outdoor activities, and their associations and personal relationships with the three ecosystems forests, peatlands, and seagrass meadows. We explained what seagrass is, as we (correctly) assumed that few participants had ever heard of it. Section “Introduction” ended with an overview and examples of the three ecosystems’ main functions. We asked whether the participants were aware of the functions, if they felt that certain functions were missing from the lists, and how they ranked their importance.

Section “Materials and methods: Focus groups and general population” focused on perceptions regarding the use of ecosystem restoration to capture and store carbon as a form of climate change mitigation. First, participants were asked how they perceived the current state of the three ecosystems and whether they thought said status was connected to climate change. Then, an information block followed. Using graphics, we briefly explained the carbon cycle, the political goal of achieving CO_2_ neutrality in Germany by 2045, and the concept of residual emissions. Next, afforestation, rewetting of peatlands, and restoration of seagrass meadows were introduced as ecosystem-based methods for removing CO_2_ from the atmosphere and compensating for residual emissions. We then gave the respondents an overview of the potential that each ecosystem holds, including information on its current CO_2_ storage efficiency, future potential for CO_2_ removal, and respective pros and cons.^2^ Subsequently, we asked the respondents how they viewed the restoration efforts; what should be done and why; who should finance the restoration; and whether the respondents would be willing to accept impacts on their own behavior and life.

In section “Results”, participants had to divide a hypothetical restoration budget of €100 between the ecosystems, so as to glean insights into their preferences for the restoration of the three ecosystems. Participants typed their answers in the chat window of the online platform but were asked to all send it at the same time on cue by the moderator to ensure that participants were not influenced by the others’ responses. We then asked them to explain their choices.

The last section was about perceptions of ecosystem governance: who should make decisions, who should participate in the discussions, and who is perceived as being trustworthy.

To identify the influence of our information provision, related experimenter demand effects, and social desirability effects in the group context, we ran a short follow-up online survey. Respondents were recruited from a commercial online panel, with recruitment quotas for gender, age, federal state, and education level to ensure representation of the online-active German population. We collected 401 responses in autumn 2022. In contrast to the focus groups, respondents received no information about afforestation, rewetting, or the restoration of seagrass meadows. Further, they were not informed about CO_2_ removal via ecosystem restoration. Like the focus group participants, they had to allocate a hypothetical budget of €100 and were then asked to explain their reasoning in a free-text question.^3^ We also ran the survey to compare it to the focus groups to explore to which extent the patterns observed in the focus group discussions appear in a broader sample.

Table 1 shows the basic demographics of the focus group participants. Roughly half of the participants were female, the median age group was 30–45 years, and roughly half of the participants held a university degree. Similarly, Table 2 displays the basic demographics of the survey participants, showing that 51.12% were female, the average age was 42.85 years (ranging from 18 to 64 years), and 27.43% had a higher education entrance certificate. Focus groups can per definition not be representative of the wider population, but the comparison shows that an important difference to the survey participants is their higher level of education.

**Table 1:** Participant IDs and demographics

ID	Gender	Age	Education	Group
GD1TN1	Male	38	University degree	urban1
GD1TN2	Male	25	A-levels	urban1
GD1TN3	Female	37	University degree	urban1
GD1TN4	Female	54	University degree	urban1
GD1TN5	Male	48	University degree	urban1
GD2TN1	Female	53	Lower secondary school	rural2
GD2TN2	Female	32	University degree	rural2
GD2TN3	Female	48	Lower secondary school	rural2
GD2TN4	Female	27	A-levels	rural2
GD2TN5	Male	62	University degree	rural2
GD3TN1	Male	27	University degree	urban3
GD3TN2	Male	44	Apprenticeship	urban3
GD3TN3	Female	25	University degree	urban3
GD3TN4	Female	23	A-levels	urban3
GD3TN5	Female	56	University degree	urban3
GD4TN1	Female	51	University degree	rural4
GD4TN2	Female	29	University degree	rural4
GD4TN3	Female	42	University degree	rural4
GD4TN4	Male	49	A-levels	rural4
GD4TN5	Male	30	A-levels	rural4
GD5TN1	Male	51	University degree	coast5
GD5TN2	Male	36	University degree	coast5
GD5TN3	Male	59	Upper secondary school	coast5
GD5TN5	Female	26	University degree	coast5
GD6TN1	Female	26	A-levels	coast6
GD6TN2	Male	45	Upper secondary school	coast6
GD6TN3	Female	34	University degree	coast6
GD6TN4	Male	44	A-levels	coast6
GD6TN5	Male	56	Upper secondary school	coast6

**Table 2:** Sample characteristics of survey participants

Characteristics	Sample	
	*N* = 401	
	Mean	Std. Dev
Female (%)	51.12	
Age (years)^a^	42.85	12.8
Higher education (%)^b^	27.43	

^a^Average age includes people between 18 and 64 years

^b^Defined as education level that is required to study at a university

The focus group discussions were transcribed and analyzed with MAXQDA, a qualitative data analysis software package. The documents were coded using an inductive approach, in the sense that categories were derived from the text without a concrete theoretical deduction (Mayring 2007). We went through the material several times to iteratively refine the codes, e.g., by identifying sub-themes within existing codes. Following the coding by one of the co-authors, we jointly discussed and partly revised the coded segments until we unanimously agreed upon the coding. Furthermore, we coded the free-text answers from the online survey where participants explained their budget allocation (Table 3).

**Table 3:** Coding of arguments for or against the allocation of restoration funds to a given ecosystem

Category	Description of argument category
Storage capacity	
Pro	High storage capacity
Contra	Argument that highlights the comparatively low storage capacity of a given ecosystem
Other ecosystem services	Promotion of other ecosystem services (besides CO_2_ storage)
Protection and restoration	
Pro	Need for protection and/or restoration, e.g., to compensate for historical interventions
Contra	No need for additional protection and/or restoration, e.g., because we are already doing enough
Attachment to ecosystem	
Pro	Positive emotional attachment to the ecosystem
Contra	Lack of emotional attachment to the ecosystem
Land use	
Pro	Low land use for restoration of the ecosystem, e.g., low impact on land use competition
Contra	High land use for restoration of the ecosystem, e.g., high impact on land use competition
Costs	
Pro	Comparatively low costs of restoring the ecosystem
Contra	Comparatively high costs of restoring the ecosystem
Research and innovation potential	Confidence in future innovation and/or research that would make the restoration easier and/or decrease costs
Other arguments	Ambiguous arguments that did not fit into any of the other categories

## Results

### Initial associations with forests, peatlands, and seagrass meadows

Participants’ spontaneous associations with the three ecosystems varied from individual words or adjectives (e.g., “*green*” (GD1TN1) or “*quiet*” (several respondents, e.g., GD4TN3)) to longer statements (e.g., “*When I want to do something good for myself, I go into the forest.*” (GD6TN1)). In total, we coded 146 associations as *positive*, *negative* or *neutral*.^4^ Here, *positive* refers to expressions that explicitly (through explanation by the speaker) or implicitly (through the semantic meaning) have a positive connotation or association with a given ecosystem (e.g., “*the scent of trees*” (GD3TN3); “*always good mood*” (GD3TN5)). Expressions that explicitly or implicitly had a negative connotation or association with a given ecosystem were coded as *negative*. This code includes both a possible threat to the ecosystem and a negative perception of the ecosystem itself (e.g., “*A lot of forests are destroyed.”* (GD2TN1); “*The area always looks a bit gloomy.”* (GD1TN3)). If a neutral attitude on the part of the speaker could be assumed, we assigned the code *neutral* (e.g., *“wildlife”* (GD4TN4); “*I also think first of the Baltic Sea and the North Sea.”* (GD3TN3)).

Associations with forests were predominantly positive and were often linked to individual recreational experiences. For example, one participant referred to “*relaxation, recreation”* (GD6TN2). We also identified environmental concerns about the forest ecosystem, referring to matters such as monoculture or increasing deforestation, indicating a certain critical awareness of environmental stressors which we could, however, almost exclusively observe for forests.

In contrast, we recorded mostly negative associations with peatlands, e.g.,:

*“[…] but a peatland can also be dangerous if you aren’t careful.”* *(GD3TN2)*

*“I never liked peatlands, I always found them a bit scary, I must say, as a kid." (GD4TN1)*

These associations partly stemmed from films or novels, in contrast to the associations with forests, which were mostly based on personal experiences. Thus, there was a greater emotional distance to peatlands (“*Peatland is not so close to my heart in terms of distance.”* (GD6TN1)). For a small number of participants who had actually had more frequent personal experiences with peatlands, we also recorded positive associations (“*[There are] insects and flowers that can’t be found anywhere else.*” (GD3TN2); “*I actually thought it was nice there.”* (GD3TN4)).

With regard to seagrass, we counted far fewer associations than for forests and peatlands, showing that the participants were generally less aware of and mostly unfamiliar with seagrass. Furthermore, the statements were predominantly neutral:

*“I can’t relate to that right now. I don’t have a picture in my mind right now.” (GD4TN5)*

Occasionally, participants had negative associations with seagrass, mostly when they thought more generally about beach wrack, which they associated with an offensive smell, though some said *“It looks nice.”* (GD2TN3). Compared to the two other ecosystems, we identified a relatively high number of *unclear* associations, which further underlines participants’ lack of familiarity with seagrass.

Like with forests and peatlands, associations did not differ systematically by place of residence. Living on the coast did not lead to more positive or fewer negative associations with seagrass. In only one of the *coast* groups did participants refer to coverage on the regional TV station about seagrass and peatlands, indicating a higher awareness of seagrass among these coastal residents.

### Perceptions of interventions in nature

The majority of participants supported the active restoration of ecosystems to increase CO_2_ removal. For example, one of the participants stressed that:

*“We have to do something, we can’t just sit back and watch what happens, we have a next generation that wants to be enjoy a decent quality of life." (GD5TN3)*

Respondents did not perceive the measures as interventions in nature per se, though they do change the landscape and affect the current ecosystems. Rather, restoration measures were perceived as restoring the previous natural state that was lost due to human interventions in the past. *“I also think it’s more like we’re making up a little bit for what we destroyed before.”* (GD3TN3) The following statement shows that ecosystems are perceived as tried and tested compared to market-based policy instruments, even though restoration could be financed via emissions trading:

*“It is always better when you come up with something natural or if you restore what has worked well somewhere or comes from nature compared to solving the problem with emissions certificates or whatnot.” (GD1TN5)*

Moreover, we found very little concern about potential economic side effects of restoration efforts, e.g., on the agricultural sector. Only a few respondents discussed trade-offs with food production. Instead, the restoration of ecosystems and the potential reduction of available farmland was repeatedly linked to a more general discussion about issues like overproduction and food waste. One participant argued: *“But first, I would say it’s not bad because too much [food] is produced.”* (GD3TN3). Only a few participants explicitly mentioned that food prices in Germany could rise and argued that higher prices might lead to more *“conscientious”* and *“careful”* consumption habits (e.g., GD6TN2).

Further, increasing CO_2_ removal capacities was not considered the only or necessarily the most important reason for restoring ecosystems; other reasons mentioned included faunal biodiversity, and—in each case by one participant only—hazard prevention and taking responsibility for future generations. However, we only received a limited number of answers concerning the relevance of restoration measures for increasing CO_2_ storage capacity. This may have been due to the complexity of the topic. In fact, some participants knew very little about the functioning of CO_2_ storage in the different ecosystems beforehand, and even after our explanations still had some difficulties understanding the subject matter.

### Relative importance of restoring the ecosystems

**Fig. 1:**
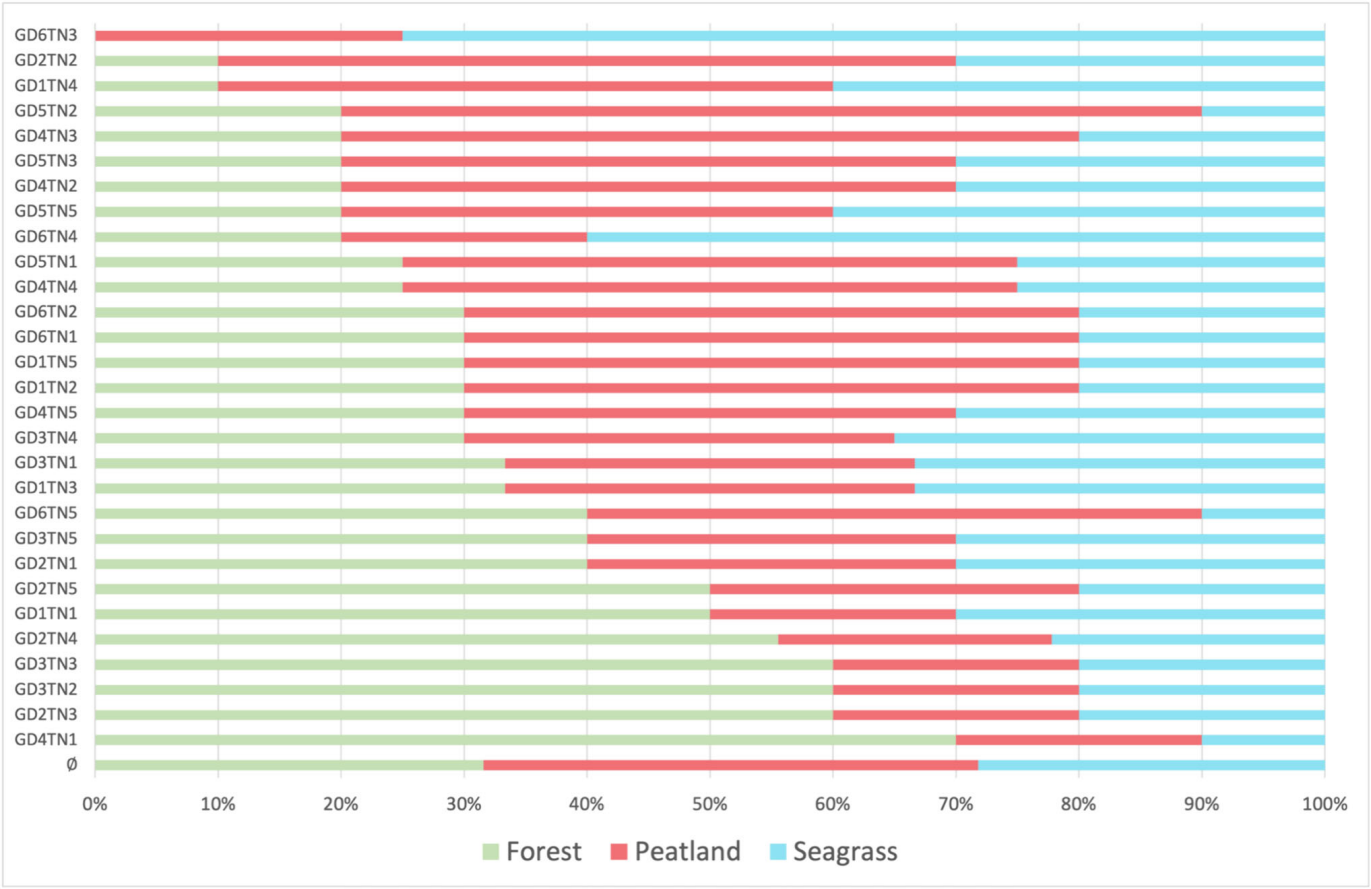
Allocation of the €100 restoration budget to forests, peatlands, and seagrass meadows (per participant)

Figure 1 shows each participant’s allocation of a hypothetical €100 restoration budget to the three ecosystems.^5^ On average, participants allocated funds relatively evenly among the three ecosystems; however, the average allocation favored peatlands (€38.47), followed by forests (€32.99) and seagrass (€27.64). If we zoom in on individual participants, the distribution varies considerably. The ecosystems’ shares ranged between 10 and ~ 75%. However, all but one participant allocated at least 10% to each ecosystem, so that none got nothing. Looking at the group level, *rural2* and *urban3* stood out by allocating on average the highest amount to forests at the expense of peatlands, while *coast5* members allocated noticeably more than average to peatlands at the expense of forests and *coast6* allocated a noticeably higher share to seagrass at the expense of forests.

When asked to explain the reasons for their choices, three principles of distributive justice were mentioned: pursuing equal distribution between all three ecosystems; ensuring that none of the ecosystems was left out; and giving the most support to the ecosystem that had suffered most in the past and was seen as most threatened.

Respondents’ explanations of why they allocated more or less money to a specific ecosystem differed in length, detail, and in the number of arguments they used. Overall, we coded 87 different arguments justifying the distribution of funds to a particular ecosystem—31 for forests, 30 for peatlands, and 26 for seagrass meadows. While we did not specify how many arguments participants should make per ecosystem, every participant was prompted to at least say something. Therefore, some respondents had one argument per ecosystem or none at all, while others had several arguments to justify the share of money they allocated to a particular ecosystem. As the share given to one ecosystem depends on the shares given to the other two, respondents’ explanations included arguments for and against funding the restoration of a specific ecosystem, i.e., to justify allocating a higher or lower share. For example, arguments about *storage capacity* were either used in favor of an ecosystem, when participants perceived its capacity as particularly high, or against an ecosystem, when its capacity was perceived as relatively low. We therefore differentiate in the coding whether the arguments were used in favor or against a given ecosystem, i.e., in favor of another ecosystem.

We coded the ecosystem-specific arguments into eight different categories, which are explained in Table 2. Participants referred to an ecosystem’s carbon *storage capacity*, the promotion of *other ecosystem services* besides CO_2_ storage, the need for *protection and restoration*, their *emotional attachment* to a given ecosystem, the *land use* for restoration, the *costs*, and the potential for cost reductions and improvements in feasibility in future, summarized under the code *research and innovation potential.*^6^

**Fig. 2:**
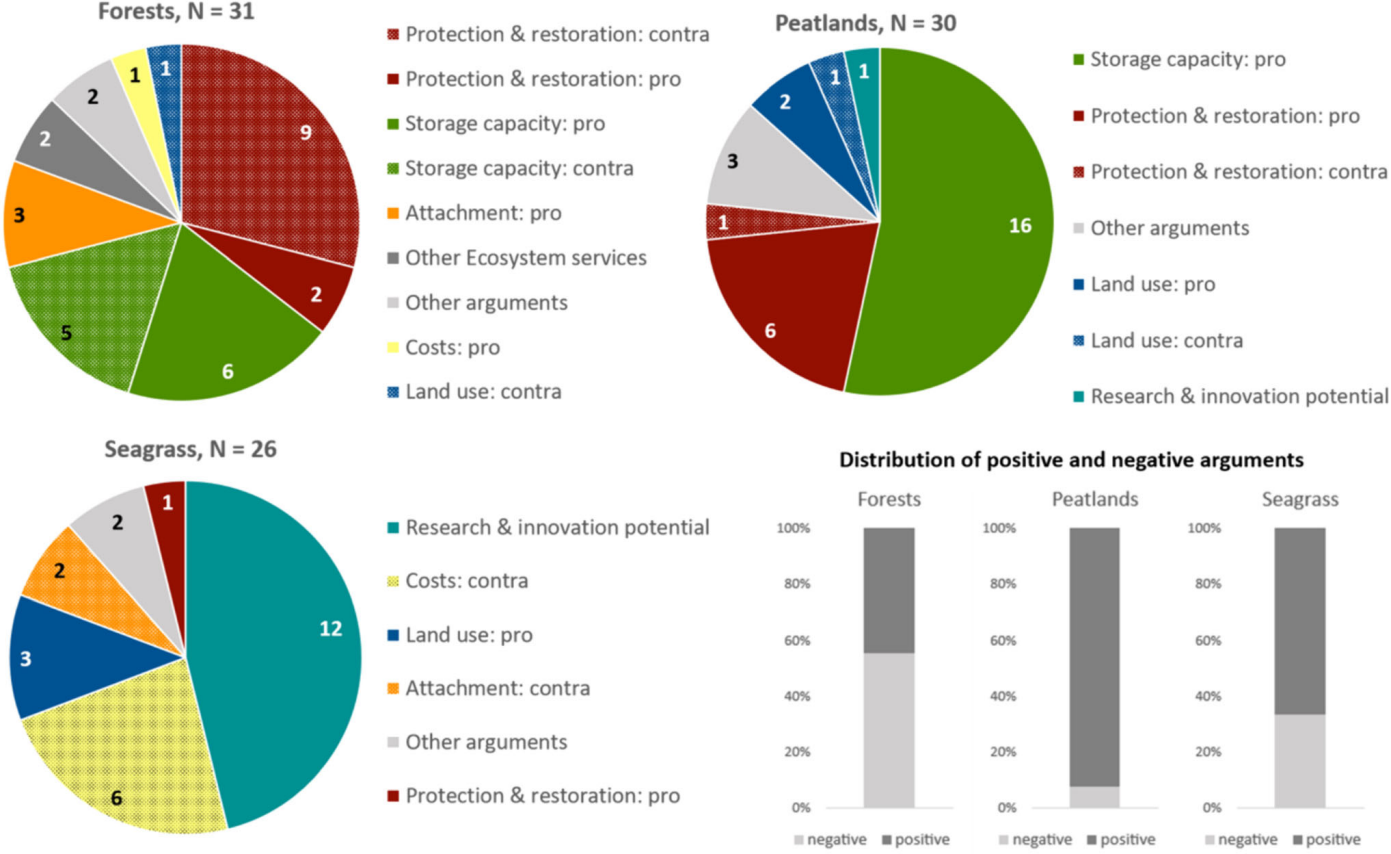
Arguments used to explain the distribution of the €100 budget and shares of positive and negative arguments (lower left panel) by ecosystem in the focus groups

Figure 2 shows the distribution of arguments by ecosystem, so as to quantify which arguments appear frequently and which arguments are particularly related to a certain ES.^7^ It also shows the ratio of arguments for and against allocating funds to a given ecosystem.

Participants used 8 different arguments in support of **afforestation**. Roughly half of the arguments were actually against afforestation. This share of counterarguments was particularly high for forests, even though on average respondents allocated a third of the budget to them. They most frequently said that there was no need for additional *protection and restoration* (*n* = 9), whereas only few felt that more should be done to protect and restore forests (*n* = 2). Several participants claimed that afforestation had the highest efficiency in terms of CO_2_ *storage* (*n* = 6), while others considered the *storage capacity* to be relatively low (*n* = 5). Forests were the only ecosystem for which participants mentioned a *positive attachment* (*n* = 3):

*“[…] and 70 euros for the forest, just because I love the forest […]" (rural4, GD4TN1)*

Only few respondents referred to *other functions*, low *costs* or the high *land use*.

For **rewetting**, we found six different arguments. Nearly all were arguments in favor of restoring the ecosystem. Participants most often emphasized the high *storage capacity* and efficiency (*n* = 16).

*“I also think that the peatlands might make the most sense […]and are relatively effective, the efficiency is relatively high.” (coast5, GD5TN1)*

In several instances, respondents also pointed out the need for *protection and/or restoration* to compensate for the harm done to peatlands, which have suffered greatly from human interventions in the past (*n* = 6):

*“[…] for peatlands, because they have suffered the most, as I said, I think about 90%, as was mentioned before, of the peatlands have been drained.” (coast6, GD6TN2)*

Only one participant claimed that the *protection and restoration* of peatlands was not necessary or less necessary compared to the other two ecosystems (*n* = 1). Arguments about *land use* and the *research and innovation potential* were rarely used.

For *seagrass restoration*, we identified five different arguments that were predominantly in favor of allocating funds. Giving funds to this ES was mainly explained by expectations about the *potential for research and innovation* (*n* = 12).

*“For seagrass, I just thought that research could find out a bit more about it.” (coast5, GD5TN5)*

*“And 30 euros for seagrass, just because I am curious to find out what this innovation will lead to. Whether it is useful at all and because I want to advance the research about it”. (rural3, GD2TN2)*

The *costs* of restoration were considered comparatively high by several participants (*n* = 6). Less often, participants emphasized the absence of *land use* conflicts (*n* = 3). Only few mentioned the need for *protection and restoration* as an argument for increasing funds or a lack of emotional *attachment to seagrass* as a reason not to allocate funds to the ecosystem*.*

### Comparison with responses from general population survey without information provision

In the general population survey, we also asked to distribute funds for restoration between the ecosystems and to explain their choice. Contrary to the focus groups, participants did not receive any additional information on the benefits, land use aspects, or costs of restoration measures. Here, we find a different distribution between the ecosystems. The 391 respondents that were in favor of public support for at least one of the ecosystems allocated on average 48 euros to afforestation, 27 euros to rewetting, and 25 euros to the restoration of seagrass meadows. Ten respondents did not want to allocate public funds to the restoration of any of the three ecosystems. This implies, that the setup of the focus groups, i.e., the (self-)selection of participants, the information provision, the group discussion, and/or the presence of a researcher, led to a shift in participants’ attention toward rewetting and seagrass restoration and away from afforestation. The general population survey provides a baseline estimate of awareness about CO_2_ storage. When asked whether they had heard before that the respective ecosystems can take up CO_2_, 91% confirmed having heard so about forests, 61% about peatlands, and 37% about seagrass meadows. 49% had never heard of seagrass meadows at all.

In the free-text answers, where survey respondents explained their allocations, we found 287 arguments that were codable. Compared to the focus group, the arguments were less diverse and more general, like *“it is important”*. We identified 4 major arguments for forests, 3 for peatlands, and only 2 for seagrass that we report here. Notably, the main argument for seagrass meadows in the focus groups, the potential for *research and innovation,* was not mentioned at all by the survey participants. We can therefore assume that it was drawn from our input in the focus groups. The lower level of familiarity with peatlands and seagrass among survey respondents compared to focus group participants is also reflected in these responses; for example, 177 of the arguments referred to forests, while only 55 mentioned peatlands and 37 seagrass meadows. However, participants in the focus groups were prompted to say something, and it takes more effort to write a balanced statement than to simply make one orally.

Personal *connectedness* was a prominent argument in favor of afforestation (*n* = 68), while the lack of *connectedness* was cited as an argument against the restoration of peatlands (*n* = 17) and seagrass meadows (*n* = 24). No one made arguments for allocating a lower share to afforestation. But *other functions* (*n* = 50), the *need for protection* (*n* = 33), and *storage capacity* (*n* = 26) were mentioned as positive aspects of afforestation. Similar to the focus group discussions (though to a lesser extent), *storage capacity* (*n* = 23) was the most important argument for peatland restoration. The *need for protection* came up as well (*n* = 15). Beyond the lack of personal *connectedness*, *other ecosystem services* were mentioned in favor of seagrass (*n* = 13). For all three ecosystems, numerous statements were *unclear* and could not be coded; for example, one participant stated that “*forests and peatlands are regional”—*without any further explanation, while another argued that “*there is only a limited amount of money at our disposal*” (forests: *n* = 41; peatlands: *n* = 36; seagrass *n* = 38 for unclear).

### Perceptions of and positions on ecosystem governance

Overall, we identified a relatively low level of trust^8^ when asking about the participants’ trust in politicians to adequately decide about the use and treatment of ecosystems in the context of climate policy. The arguments can first of all be clustered around low levels of perceived transparency of political processes and allegations regarding corruption and politicians pursuing their own interests (*n* = 14), which were mostly general and not directly related to the specific context of ecosystem restoration.

A second prominent discourse in the context of political trust was the relevance of expertise, more specifically assumptions about a lack of expertise among political actors. For example, one participant noted:

*“I believe that first of all a fundamental problem is that our politicians are not experts, so you can only hope that there are as many experts as possible behind them." (GD5TN1)*

Four participants expressed an increase in political trust with respect to environmental expertise since the Green party had joined Germany’s coalition government, shortly before the focus groups in late 2021.

Moreover, we identified a higher level of trust in local politicians compared to politicians at other levels of governance. Participants felt that local politicians were closer to the concerns of the people, especially when compared to federal and especially EU politicians. For example: “*They [local politicians] are of course closest to the people”*. (GD5TN1) On the other hand, trust in politicians and political processes at the EU level was particularly low. Distrust in them was specifically articulated 9 times. In the context of ecosystem restoration, some participants also alluded to the collective action problem of freeriding, which they considered highly problematic at the international level (EU). According to one participant:

*“But I would say, what good does it do if we Germans, let’s say, really do something here and all the other countries do nothing? Then, in the end, it would be just as if no one had done anything.” (GD4TN4)*

In general, there was a clear demand for the participation of non-state actors in the governance of ecosystem-based solutions for CO_2_ removal. Suggestions for specific groups of non-state actors were somewhat heterogeneous, but overall, we saw a high demand for (scientific) experts, such as biologists or engineers (*n* = 19). On the other hand, numerous participants stressed the importance of involving property owners and other locally affected groups (*n* = 19).

A further, but less frequent argument for public participation was the democratic principle of universal representation. Interestingly, several participants even expressed their skepticism toward broader public participation due to a lack of expertise. For example, one participant argued: *“I don’t think you should just let citizens decide who may not know that much about it. I think that expertise is called for.”* (GD3TN4) When asked about their own willingness to get involved in political decision-making on ecosystem restoration, only about half of the participants responded at all. Out of these, 11 participants indicated they would be willing to get involved.

When we asked participants if they considered ecosystem restoration legitimate if it meant having to sacrifice farmland areas, the majority responded in the affirmative. Expropriation was explicitly endorsed; the issue of compensation was occasionally mentioned but not of major concern. Related issues of distributional justice were only discussed in passing. For example, one participant stressed the need to avoid that: *“the small farmers suffering from it who perhaps have worked there all their lives.”* (GD5TN5).

While largely supporting the restoration of farmland, some participants voiced concerns about national food security. So one participant argued: *“One important question is, would our food supply still be secure?”* (GD2TN5).

When asked who should pay for the restoration, the majority felt that all taxpayers should pay. Some participants argued that the specific amount of money to be paid should depend on actors’ CO_2_ emissions. According to one:

*“And I would say, starting, of course, with the entrepreneurs who have the big bucks and also especially the trucking companies that also have a lot of trucks, and so on". (GD2TN3)*

Others felt that the amount should depend on the level of income; thus, these participants used the polluter-pays principle and the capabilities approach as arguments for burden sharing.

## Discussion

Based on six in-depth focus group discussions in Germany, we analyzed public perceptions of restoring forests, peatlands, and seagrass meadows including the priorities people make when choosing between the ecosystems and their perceptions of ecosystem governance.

Forests as “charismatic ecosystems” were highly popular; peatlands evoked negative associations, and seagrass was largely unknown. Beyond charisma this was also driven by the level of familiarity and connectedness with the ecosystems. By and large, this is in line with previous research findings (e.g., Byg et al. 2017; Ruiz-Frau et al. 2018; Rathmann et al. 2020) but it also highlights the importance of geographical differences. For example, Scottish study participants (Byg et al. 2017; Faccioli et al. 2020) show markedly positive associations with peatlands probably because they are considered a typical Scottish landscape and people are familiar with it.

In participants’ reasoning about the weighing of support for the restoration of the ecosystems, we found quantitatively and qualitatively more nuanced arguments about afforestation, compared to peatland rewetting and seagrass restoration. We attribute this to participants’ higher level of knowledge about forests. For example, participants talked about issues such as monoculture and deforestation. There were many instances in which participants expressed personal connectedness, recreational use, and perceptions of forests as being charismatic. This positive emotional attachment and the individuals’ use value also seems to lead to a preference for afforestation. We found limited associations, and non-existent or negative emotional attachment with seagrass and peatlands, in addition to little or no knowledge about these two ecosystems. Arguments about personal use and a positive *emotional attachment*, as well as the promotion of *other ecosystem services*, were solely used for forests.

Even when they do not know a lot about an ecosystem, participants still find it important to finance restoration. They go beyond individualistic motives and used justice-based arguments when explaining their support for rewetting or the replanting of seagrass. They felt that none of the three ecosystems should be entirely excluded from restoration, that these ecosystems should be protected and restored for their own sake, and that past harm should be compensated for. Similarly, Martin-Ortega et al. (2017, p. 15), who investigated criteria for where to restore peatlands, identified the criterion where *“little is left”*. Discussing after-use potentials of harvested peatlands, Collier and Scott (2008, p. 450) also found the motive that it is *“[…]only fair to let them live again.”* More concrete issues around restoring especially land-based areas such as transforming agricultural land, local public opposition, or impacts on food prices were not prominent in the discussions.

The focus group discussions offer nuanced insights into discourses and patterns of perceptions, but the small number of participants, the recruitment based on prior interest in the topic, and the (self)selection into the study limits the generalizability of results (Nyumba et al. 2018). Furthermore, the information we as researchers provided at the focus groups is more extensive than what most people in real life will normally have available, it can cause experimenter demand effects, and social desirability within the group to appear more likable. The focus group results might therefore lead to an overestimation of the positive views on ecosystem restoration. To put our findings into perspective, we ran a general population survey where respondents had not received any information on ecosystem services, trade-offs, or the costs of restoration. They responded anonymously without being prompted by a moderator to say anything.

Overall, the general population survey corroborates the results from the focus groups. There is widespread support for ecosystem restoration and forests are popular. A notable difference is that while peatlands received the largest share of funds in the focus groups, forests did so in the survey. The justifications for the allocation of funds between ecosystems were less diverse and more general in the survey responses. Afforestation was more prominent while focus group participants used arguments that reflected the information they received and the group discussions. For example, peatlands’ potential to sequester and store CO_2_ was an aspect many focus group participants used to justify the allocation of money to rewetting and apparently weakened the focus on forests in the discussions. In the general population survey on the other hand, about 39 percent stated that they were not aware that peatlands stored CO_2_. From our information on seagrass restoration, focus group participants had clearly taken up the argument about the potential of future research and innovations to make it cheaper and easier. Though this argument reflects information we gave to the participants, Fernández et al. (2022) also observed an increasing positive perception of seagrass along with an increasing newspaper coverage of research activities on seagrass restoration. This implies in line with previous research (Schaafsma et al. 2017) that information provision can alter initial attitudes and preferences for restoration interventions.

Turning to the perception of ecosystem governance, most of the focus group participants called for the selective political participation of non-state actors but not of the broader public. This stands in contrast to the majority of policy recommendations (see also Bennett 2016) that involving the public will foster successful and legitimate ecosystem management. Instead, they saw (scientific) expertise on the one hand and direct (financial) affectedness on the other as relevant criteria for participation in the decision-making process. Many participants voiced distrust in politicians and their ability to make good decisions about ecosystem restoration. The apparent concerns about public participation were the perceived complexity of ecosystem management, the need for scientific expertise, and the lack of expertise among the general public, as similarly documented in prior research on genetics (Kerr et al. 2007). Whether this stance was due to growing democratic disengagement, as observed in advanced democracies in several other studies (Hay 2007; Grasso et al. 2019), is beyond the scope of this work. In addition, the presence of researchers at the group discussions might have further influenced participants focus on expert involvement. But overall, these results do not necessarily undermine the importance of public participation in general. Instead, the insights call for a further differentiation of policy recommendations regarding public participation: the complexity of the issue area as well as existing expertise in the public (respectively the lack of it) should be taken more closely into consideration. As we also found a low level of trust in politicians and their expertise on the matter, it calls for including (scientific) experts such as biologists, engineers, or NGOs to compensate for the perceived lack of expertise. Moreover, depending on the specific issue area and its complexity scientific expertise could be included in engagement processes to enable meaningful public participation.

Further analyzing the issue of political trust, our findings on the particularly low trust in politicians and politics at the EU level contrast, however, with the empirical results of a Europe-wide survey on public perceptions about marine environmental impacts and governance finding a relatively higher trust in EU level politics, as presented by Gelcich et al. (2014), and those of the study by Gkargkavouzi et al. (2020) on political trust in three Greek cities. Our results are rather in line with the compensation model (Sánchez-Cuenca 2000; Muñoz et al. 2011), which predicts that comparatively higher trust in national and subnational political capacity—as here in ecosystem restoration management in Germany—leads to lower levels of trust in European institutions and vice versa, due to a comparative evaluation of performance.Here we have to consider that, despite the perceived public criticism, Germany can still be seen as one of the main leaders of European environmental and climate politics (Simonis 2017). The higher level of trust in local politicians compared to those at all other political levels is again justified by concrete knowledge or expertise on local matters.

## Conclusion

Our study looks at the support or opposition among the public that would not necessarily be affected by restoration projects as one aspect of socio-political acceptability. In future, it will be additionally valuable to pursue a spatially explicit approach when considering specific ecosystems, as residents will mainly be affected by changes in local ecosystem services. This could also include the restoration of other ecosystems and inform policy initiatives like the action program for natural climate protection in Germany (Deutscher Bundestag 2023), the planned EU Nature Restauration Law, or the Kunming-Montreal Global Biodiversity Framework (United Nations Conference of the Parties 2022).

Summarizing our insights, we can conclude that the importance of scientific expertise was highlighted in many of the responses in the focus groups: from the questions on political trust, to attitudes toward the participation of non-state actors, to the reluctance to participate personally. Furthermore, trust in science and innovation was relevant not only for the perception and evaluation of ecosystem governance, but also in the context of the allocation task in the focus group discussions, as one of the major arguments for funding seagrass restoration was the confidence that future research and innovations would provide technical solutions that increase the feasibility of large-scale seagrass restoration.

Overall, our findings suggest that there is a public demand for greater delegation of decision-making to scientific experts in an area of high complexity such as this one, and thus potentially also for greater political engagement of scientists. In this context, a stronger cooperation between politics and science could be seen as a useful tool to increase acceptability of and compliance with (future) policy regulations for ecosystem restoration, which is of growing political relevance not only in Germany but globally. However, while such political engagement of scientists opens up new opportunities for knowledge-based to pressing political challenges in the context of climate change, it also carries the risk of increasing tendencies toward a technocratic system that potentially undermines democratic principles. Consequently, to strengthen this type of policy-science cooperation, concrete mechanisms need to be developed to ensure compliance with the crucial criteria of throughput legitimacy, namely transparency, accountability, and inclusiveness (Schmidt 2013).

## Footnotes

1 We wanted to ensure that participants have had at least a minimal prior exposure to the German coast, to facilitate more engaged discussions.

2 During the focus group discussions, specific sources of the information presented were not disclosed to participants to not distract from the content. The sources were available upon request, but participants did not ask about their origins.

3 The online survey was informed by the focus group discussions and included additional follow-up questions for quantitative analysis not covered in this paper.

4 Additionally, ambiguous statements that could bear either a negative or positive connotation were coded as *unclear*. An example would be: *“I would also tend to say that there is maybe something a little bit mystical about it, a little bit of mystery.”* (GD2TN4) Here, we assumed that *“mystical”* does not have a neutral meaning, but it was unclear whether the connotation was positive or negative.

5 The question reads as follows: *“Imagine you could invest €100 in afforestation, the rewetting of peatlands and the restoration of seagrass meadows. How would you divide these €100? Note: The €100 cannot be spent on anything else”.*

6 Arguments that could not be clearly assigned due to their ambiguity were coded as *“other arguments”.*

7 If a participant indicated that his or her explanation resembled that of another participant, we assigned the same code to him or her.

8 Following Strandberg et al. (2021, p. 5) we define political trust as follows: “Political trust entails the citizens’ confidence that political institutions and procedures function according to positive expectations”.
